# Metabolite Profiling of Chestnut (*Castanea crenata*) According to Origin and Harvest Time Using ^1^H NMR Spectroscopy

**DOI:** 10.3390/foods11091325

**Published:** 2022-05-02

**Authors:** Ja Myung Yu, Miso Nam, Min-Sun Kim

**Affiliations:** Food Analysis Research Center, Korea Food Research Institute, Wanju 55365, Korea; j.m.yu@kfri.re.kr (J.M.Y.); msnam@kfri.re.kr (M.N.)

**Keywords:** chestnut (*Castanea crenata*), metabolites profiling, ^1^H nuclear magnetic resonance (NMR), origin, harvest time

## Abstract

Chestnuts are an important food crop commonly used as a food ingredient due to their nutritional properties and potential health benefits. In Korea, chestnuts have been crossbred to develop cultivars with insect resistance and high productivity, producing multiple chestnut varieties. This study classified 17 *Castanea crenata* cultivars produced in Korea according to origin and harvest time and determined the metabolites in chestnut kernels using ^1^H nuclear magnetic resonance spectroscopy. The 17 *C. crenata* cultivars were divided into four groups based on their geographic origin: Korean native, Korean hybrid, Japanese native, and Japanese hybrid. The cultivars were also divided into three groups depending on their harvest period: early-ripening cultivar, mid-ripening cultivar, and late-ripening cultivar. The partial least squares-discriminant analysis score plot revealed differences among the groups. Identified metabolites, including amino acids, organic acids, and sugars, contributed to discriminating the origin and harvest time of the *C. crenata* chestnut kernels. Significant differences were observed, mainly in amino acids, which suggests that the composition of amino acids is one factor influenced by both the origin and harvest time of *C. crenata*. These results are useful to both growers and breeders because they identify the nutritional and metabolic characteristics of each *C. crenata* cultivar.

## 1. Introduction

The chestnut, belonging to the Fagaceae family, is cultivated globally, including the Korean and Japanese chestnut (*Castanea crenata* Siebold & Zucc.), Chinese chestnut (*Castanea mollissima* Blume), European chestnut (*Castanea sativa* Miller), and North American chestnut (*Castanea dentate* (Marshall) Borkh.) [1,2,3]. According to the Food and Agriculture Organization, chestnut production continuously increased in Asia until 2014, and Korea is the second-largest producer of chestnuts in Asia after China [4]. In Korea, more than 50,000 tons of ripe chestnuts were produced from September to October every year until 2020 [5]. Chestnut is popular in Korea for its rich taste and high nutritive value [6]. Compared to other fruits, chestnut has a higher starch content and a lower moisture content, and it also contains proteins, amino acids, dietary fiber, vitamins, and minerals [7]. It is mainly prepared by roasting in winter or it consumed as a canned food and is used in traditional customs, such as wedding ceremonies and other ancestral rites. The inner skins are used as cosmetic ingredients [8,9].

In 1958, the chestnut gall wasp (*Drycocosmus kuriphylus* Yasumatsu), which damages and kills chestnut trees, appeared in Korea and spread across the country. As a result, many native Korean chestnut trees were harmed. Since then, large-scale crossbreeding of chestnut trees has been carried out to develop cultivars with resistance to insects and cold, high productivity, and large nut size [10]. The cultivars commonly grown in Korea are related to the Japanese chestnut and are derived from intra-hybridization or individual selection [11,12]. Currently, cultivars originating from Japan or Korea, such as Daebo, Okkwang, Ginyose, Tanzawa, Tsukuba, and Riheiguri, are widely cultivated in Korea. However, little information is available regarding the metabolic characteristics of these varieties [13,14].

Food qualities, such as fragrance, taste, appearance, shelf-life, and nutritional content, are determined by biochemical compositions and are reflected in the metabolite profiles [15,16]. Metabolites represent the intermediates or final products of cellular regulatory mechanisms, and they can be considered as the response of the biological system to environmental changes [17]. Because plant materials vary chemically, the same cultivars can have different chemical components due to genotype variations, environmental factors, time of harvesting, and post-harvesting factors [18]. The investigation of differences in metabolite profiling across cultivars may provide useful information for the comprehensive evaluation of the nutritional value of chestnuts, contributing to the improvement of new varieties or the selection of chestnut varieties for specific uses.

Metabolomics has proven to be a powerful tool for the extensive analysis and identification of plant and food compositions [19]. Nuclear magnetic resonance (NMR) spectroscopy gives excellent metabolite profiles by taking advantage of this technique’s high reproducibility, non-selective analysis, and complete analysis of the molecular components of a complex matrix [20]. Recently, metabolomics studies using ^1^H NMR spectroscopy or mass spectrometry have also been applied to food science [17,21]. There are not many metabolomics studies on chestnuts, and most of the previous studies have been on chestnut shells [22,23]. No metabolite profiles of *C. crenata* chestnut kernel extracts using NMR spectroscopy have been reported to date. In this study, we performed the metabolic profiling of 17 *C. crenata* cultivar kernels using ^1^H NMR spectroscopic analysis to measure the metabolite level of several metabolite classes. In addition, 17 *C. crenata* cultivars were classified according to their origin and harvest time, and metabolic profiling results were submitted for statistical multivariate analyses to determine the metabolic characteristics according each category. This research provides information on the features of chestnut cultivars according to their origin and harvest time that is useful to both growers and breeders.

## 2. Materials and Methods

### 2.1. Standards and Reagents

A 99.8% methanol-d_4_ and 99.9% deuterium oxide were purchased from Cambridge Isotope Laboratories, Inc. (Andover, MA, USA), 3-(trimethylsilyl)propionic-2,2,3,3-d4 acid (TSP), and hydrochloric acid were obtained from Sigma-Aldrich (St. Louis, MO, USA), and sodium hydroxide was obtained from Junsei Chemical (Tokyo, Japan). All chemicals were of analytical grade.

### 2.2. Sample Collection

*C. crenata* chestnuts were commercially purchased, approximately 4 kg per sample, from local markets in South Korea. A total of seventeen *C. crenata* chestnuts were collected between September and November 2020. All chestnut varieties were produced in Buyeo-gun or Gongju-si, Chungcheongnam-do, South Korea. The sample information of *C. crenata* are shown in Table 1. *C. crenata* cultivars were classified into four groups depending on the *C. crenata* chestnut cultivar origin: Korean native or crossbred between Korean native (KN), Korean hybrid crossbred with other origins (KH), Japanese native or crossbred between Japanese native (JN), and Japanese hybrid crossbred with other origins (JH). Also, the chestnut varieties were divided into three types according to their optimal ripening time: early-ripening cultivar (ERC), mid-ripening cultivar (MRC), and late-ripening cultivar (LRC).

To prepare the kernels, the outer shell and inner shell from the chestnuts were separated automatically using a chestnut skin removal machine. They were stored at −80 °C before freeze-drying. They were put in a freeze dryer (FDCF-12003, Operon, Korea) and freeze-dried for 72 h under conditions with a condenser temperature of −50 °C and a pressure of 10 mTorr. Dried kernels were ground to a fine powder and stored at −80 °C until the analysis.

### 2.3. Sample Preparation

The metabolite extraction step was performed following a method described by Jung et al. [24] but was slightly modified. Approximately 50 mg of dried sample was extracted in 500 μL of methanol-d_4_, 400 μL of a 0.2 M sodium phosphate buffer in D_2_O (pH 7.0), and 100 μL of 6 mM TSP. D_2_O was used as a field frequency lock signal and TSP was an internal standard with a chemical shift reference (δ) of 0.0 ppm. The mixtures were vortexed and sonicated for 20 min, and then adjusted to pH 7.0 ± 0.1 using 0.2 M NaOH and 0.2 M HCl solutions. Afterwards, they were centrifuged at 12,000 rpm at room temperature for 20 min. The upper layer was transferred into 5 mm diameter NMR tubes and stored at 4 °C until analysis.

### 2.4. ^1^H NMR Analysis

^1^H NMR spectra were recorded on a Bruker AbanceHD 800 MHz FT-NMR Spectrometer (Bruker BioSpin Co., Billerica, MA, USA), operating at 298 K, using a 5 mm triple resonance inverse cryoprobe with Z-gradients. The NOESY pulse sequence was applied to suppress the residual water signal. For each sample, 64 transients were collected in to 64,000 data points using a spectral width of 16,393.4 Hz with a relaxation delay of 2.0 s, and an acquisition time of 2.0 s. A line-broadening function of 0.5 Hz was applied to all spectra for Fourier transformation. All NMR spectra were phased and underwent baseline correction. Signal assignments for representative samples were facilitated by using two-dimensional (2D) total correlation spectroscopy (TOCSY), 800 MHz NMR database of Chenomx NMR Suite Version 8.6 (Edmonton, AB, Canada), and spiking experiments [25]. In addition, the Chenomx NMR Suite Version 8.6 software was used to quantify the metabolites in *C. crenata*. This program compares the concentration of a known reference signal (TSP) with signals derived from a library of compounds to determine the concentrations of individual metabolites [26].

### 2.5. Statistical Analysis

Partial least squares-discriminant analysis (PLS-DA) were performed with a unit variance scale using SIMCA-P+, version 16.0 (Umetrics, Umeå, Sweden). PLS-DA was performed to maximize the separation between samples. The PLS-DA was described as a regression extension of the principal component analysis (PCA) that provides the maximum covariance between the measured data (X) and the response variable (Y) [27]. R^2^ and Q^2^ values describe the quality of the models. R^2^ represents the goodness of fit and is defined as a proportion of the variance in the data described by the models. Q^2^ represents the predictability and is defined as the proportion of variance in the predictable data [27]. Also, to verify the significant differences in the quantified metabolites between more than two groups, the nonparametric Kruskal–Wallis test followed by Dunn’s post hoc test was performed using GraphPad Prism, version 5 (San Diego, CA, USA).

## 3. Results and Discussion

### 3.1. Metabolite Profiling of C. crenata by ^1^H NMR Spectroscopy

**Figure 1:**
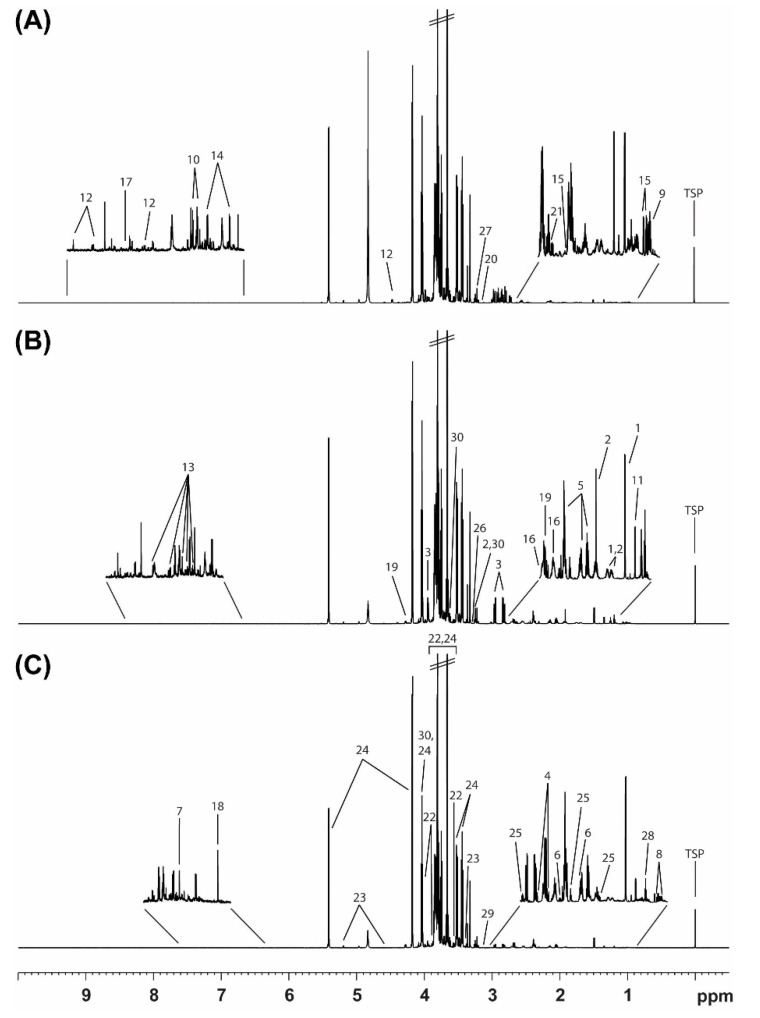
800 MHz ^1^H NMR spectra of pulp of *Castanea crenata* harvested in Chungcheongnam-do. (**A**) Okkwang (Korean native and mid-ripening cultivar), (**B**) Ginyosi (Japanese native and late-ripening cultivar), (**C**) Moriwase (Japan hybrid which crossbreeds with other origins and early-ripening cultivar).(1, alanine; 2, arginine; 3, asparagine; 4, aspartate; 5, glutamate; 6, glutamine; 7, histidine; 8, isoleucine; 9, leucine; 10, phenylalanine; 11, threonine; 12, trigonelline; 13, tryptophan; 14, tyrosine; 15, valine; 16, citric acid; 17, formic acid; 18, fumaric acid; 19, malic acid; 20, malonic acid; 21, succinic acid; 22, fructose; 23, glucose; 24, sucrose; 25, 4-aminobutyrate; 26, betaine; 27, choline; 28, ethanol; 29, ethanolamine; 30, myo-inositol).

We analyzed the kernels of 17 *C. crenata* chestnut cultivars produced in Buyeo-gun or Gongju-si, Chungcheongnam-do, using ^1^H NMR spectroscopy to determine metabolite differences. To minimize the impact of the cultivation environment, we selected *C. crenata* chestnuts produced in Buyeo-gun or Gongju-si, which are geographically close and have similar environments, in the Chungcheongnam-do Province. Figure 1 shows representative 800 MHz ^1^H NMR spectra of Okkwang (KN and MRC), Ginyosi (JN and LRC), and Moriwase (JH, which crossbreeds easily, and ERC), which are three cultivars of the *C. crenata* chestnut. The most dominant part of each ^1^H NMR spectrum was in the carbohydrate region (3.2–5.4 ppm), whereas the aromatic regions (6.9–8.5 ppm) had very low intensities. The spectral resonances of metabolites were assigned according to the 800 MHz library from Chenomx NMR Suite (version 8.6), 2D TOCSY NMR spectra, and spiking experiments.

The analysis of the *C. crenata* chestnut kernel extracts by ^1^H NMR spectroscopy detected the essential primary metabolites. The chemical shifts of the identified metabolites are listed in Table 2. A total of 30 metabolites were quantified, including 15 amino acids (alanine, arginine, asparagine, aspartate, glutamate, glutamine, histidine, isoleucine, leucine, phenylalanine, threonine, trigonelline, tryptophan, tyrosine, and valine), six organic acids (citric acid, formic acid, fumaric acid, malic acid, malonic acid, and succinic acid), three sugars (fructose, glucose, and sucrose), and six other compounds (4-aminobutyrate, betaine, choline, ethanol, ethanolamine, and myo-inositol).

### 3.2. Metabolic Characterization Depending on C. crenata Chestnut Cultivar Origin

As most chestnut cultivars cultivated in Korea originated in Japan or Korea, differences in metabolites across the varieties were first examined according to their origin. Although the morphological characteristics of the Korean and Japanese varieties are similar, a previous study suggested that the Japanese *C. crenata* and the Korean *C. crenata* might have separate evolutionary histories, according to a phenogram constructed based on polymorphisms [11]. These genetic differences may affect their metabolite contents. We classified the *C. crenata* cultivars into four groups: KN, KH, JN, and JH. The average concentrations of all metabolites identified in *C. crenata* chestnut kernels for each group are presented in Table S1.

**Figure 2:**
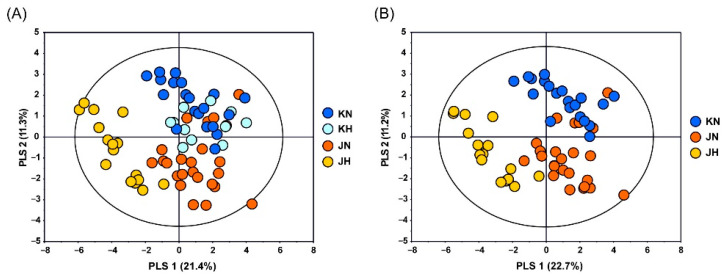
(**A**) PLS-DA score plot (R^2^X = 0.441, R^2^Y = 0.516, Q^2^ = 0.356) of quantified metabolites in KN, JN, KH, and JH *C. crenata* chestnut show the pattern in which KH is included in KN. (**B**) PLS-DA score plot (R^2^X = 0.339, R^2^Y = 0.609, Q^2^ = 0.521) of quantified metabolites shows the separation among KN, JN, and JH *C. crenata* chestnut. Letters on the legend represent: KN, Korean native or crossbred between Korean native; KH, Korean hybrid which crossbreeds with other origins; JN, Japanese native or crossbred between Japanese native; JH, Japanese hybrid which crossbreeds with other origins.

PLS-DA was performed on the concentrations of the identified metabolites to discriminate the four groups of *C. crenata* chestnuts, as shown in Figure 2. Good discrimination between Korean and Japanese cultivars was achieved according to the second component (PLS-2), which had a goodness of fit of R^2^Y = 0.516 and predictability of Q^2^ = 0.356 (Figure 2A). No significant differences were observed between the Korean cultivars categorized as KN and KH, but distinct differences were identified between the Japanese cultivars categorized as JN and JH. In particular, the JH group was distinct from the other three groups based on the first component (PLS-1) of the PLS-DA score plot, indicating that the JH group features different metabolic patterns from the other groups. Because the KH group displayed no significant difference from the KN group, and PLS-DA was performed for three groups, except the KH group, to detect more distinct metabolite patterns among the three groups. The PLS-DA score plot showed good separation among the three groups with a goodness of fit of R^2^Y = 0.609 and predictability of Q^2^ = 0.521 (Figure 2B). Unlike the other three groups, the JH group was hybridized with the Chinese chestnut (*Castanea mollissima* Blume) or the Pyongyang chestnut (*Castanea bungeana* Blume), which are different species from *C. crenata*. These results show that the cultivars in the JH group, which are produced by crossbreeding with other species, have a large difference in metabolite content compared with the other cultivars cultivated in Korea, and the origins of hybrid species could affect the metabolite levels in *C. crenata* chestnut kernels.

**Figure 3:**
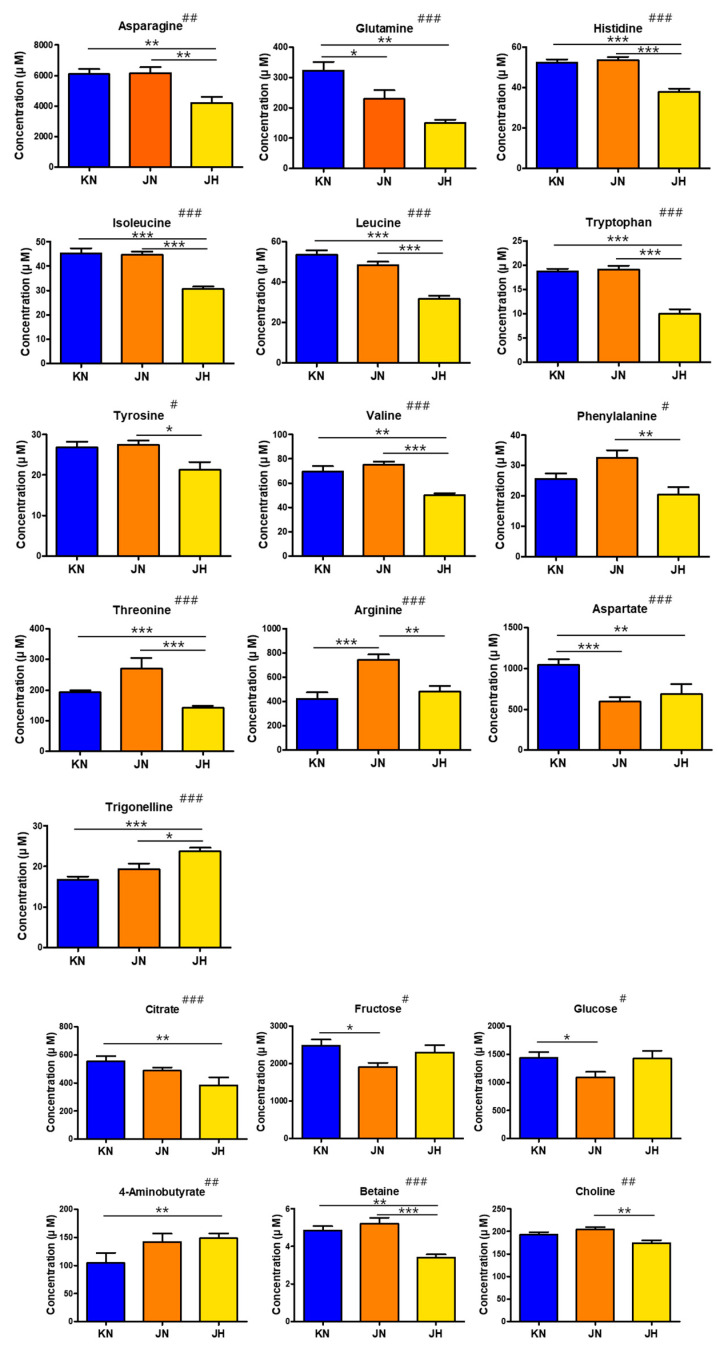
Quantification of metabolites in KN, JN, and JH of *C. crenata* chestnut. Kruskal–Wallis tests yielded significant differences determined by Dunn’s multiple comparison post hoc tests using GraphPad Prism 5.0. Significant differences in Kruskal–Wallis tests are represented as # *p* < 0.05, ## *p* < 0.01, and ### *p* < 0.001 and by Dunn’s multiple comparison post hoc tests are represented as * *p* < 0.05, ** *p* < 0.01, and *** *p* < 0.001. Error bars indicate means ± standard deviation. Letters on the horizontal axis represent: KN, Korean native or crossbred between Korean native; KH, Korean hybrid which crossbreeds with other origins; JN, Japanese native or crossbred between Japanese native; JH, Japanese hybrid which crossbreeds with other origins.

As a result of the univariate analysis for assessing the differences in metabolite levels among the groups, 19 of the 30 identified metabolites showed significant concentration differences between KN, JN, and JH, which is evident in the PLS-DA plots (Figure 3).

Of the 19 metabolites with significant differences, 13 were amino acids, which indicates that the amino acid profile is one of the main factors that can be used to determine the origins of *C. crenata*. Amino acids play an important role in cellular metabolism [28], and high levels of glutamic acid and aspartic acid in food influences taste and flavor [29]. The most abundant amino acids identified in *C. crenata* chestnut kernels were asparagine and glutamate, but most of the essential amino acids were present. In particular, the JH group was characterized as having reduced amino acid levels compared with the other two groups, except for trigonelline. The amino acids that showed significant differences between the KN and JN groups were glutamine, arginine, and aspartate, whereas the concentrations of other amino acids were similar between these two groups.

Sugars, including fructose, glucose, and sucrose, were identified in *C. crenata* chestnut kernels. The sugar composition alters the sensory characteristics of chestnuts by making them sweeter; therefore, the sugar profile represents a commercially important quality indicator [30,31]. Sugar variance has also been used for the inter-cultivar discrimination of chestnuts [32]. Sucrose was the most abundant compound among the metabolites identified in *C. crenata* chestnut kernels, with average concentrations between 38,943 and 42,280 µM in each group. This finding was similar to previous analyses reporting a high sucrose content in chestnuts [30] but no significant difference in sucrose concentrations was identified between the groups. The lowest fructose and glucose levels were detected in the JN group, which were significantly different from those in the KN group but not those in the JH group. Similar to our results, previous studies also reported the highest fructose contents for the Pyeonggi (KN group) and Riheiguri (JH group) cultivars [13]. Our results showed that fructose and glucose could be used as discriminatory factors to distinguish between the KN and JN groups.

Citric acid showed a significant difference in concentration between the KN and JH groups, and its concentration was the lowest in the JH group. Malic, citric, and ascorbic acids were the major organic acids found in the chestnut kernels, and some differences were observed among the organic acid compositions of the chestnut varieties [33]. The properties and levels of organic acids are key factors influencing the flavors of fruits and vegetables. In addition, organic acids can protect against various diseases because of their antioxidant activities [34].

Choline and betaine had significantly lower levels in the JH group compared with the JN group. Choline, which is abundant in plants, is an important nutrient that acts as a biosynthesis precursor of phospholipids and the neurotransmitter acetylcholine [35]. Betaine tends to accumulate in the cytoplasm and intercellular fluids, where it exerts protective functions on the structures of proteins, nucleic acids, and cell membranes, in response to abiotic plant stresses such as the reduced availability of water, high soil salinity, hypoxia, cold, and freezing [35].

The 4-aminobutyrate (GABA) level was the lowest in the KN group, and the difference was significant compared to the JH group. GABA can affect fruit storage or seed germination by changing the metabolism of nitrogen and carbon [36] and contributes to plant development and stress adaptation [37,38,39]. In addition, GABA modulates anion flux in plants through its role in signal transduction, which regulates plant physiology [40].

### 3.3. Metabolic Characterization Depending on Harvest Time of C. crenata Chestnut Cultivars

**Figure 4:**
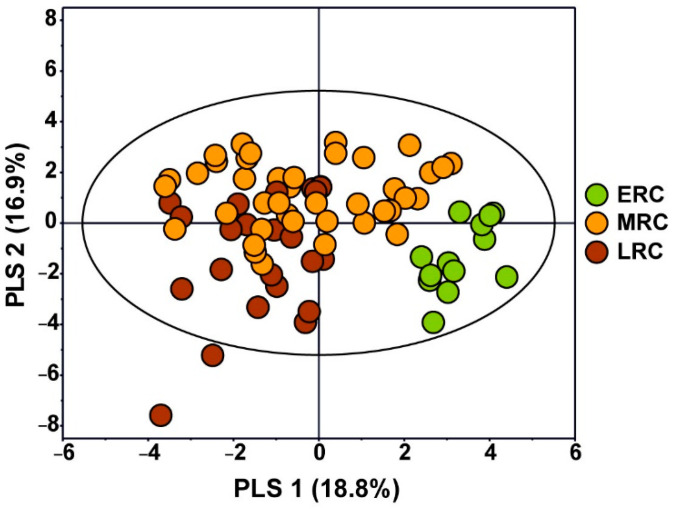
PLS-DA score plot (R^2^X = 0.357, R^2^Y = 0.417, Q^2^ = 0.324) of quantified metabolites in ERC, MRC, and LRC of *C. crenata* chestnut. Letters on the legend represent: ERC, early-ripening cultivar; MRC, mid-ripening cultivar; LRC, late-ripening cultivar.

The chestnut varieties were divided into three types according to the optimal ripening time: ERC, MRC, and LRC. The optimal ripening time for ERCs ranges from the end of August to early September, the optimal ripening time for MRCs is mid-September, and the optimal ripening time for LRCs ranges from the end of September to early October. The optimal ripening time for each chestnut variety determines the harvest time, which affects metabolite concentrations. The average contents of all identified metabolites in *C. crenata* chestnut kernels for each group are shown in Table S2. In Figure 4, the PLS-DA score plot shows the separation among the three groups using three components and has R2Y and Q2 values of 0.417 and 0.324, respectively. In particular, the ERC group was the most strongly discriminated from the other groups using PLS-1. These results showed that the harvest time affects the concentrations of metabolites in *C. crenata* chestnut kernels.

**Figure 5:**
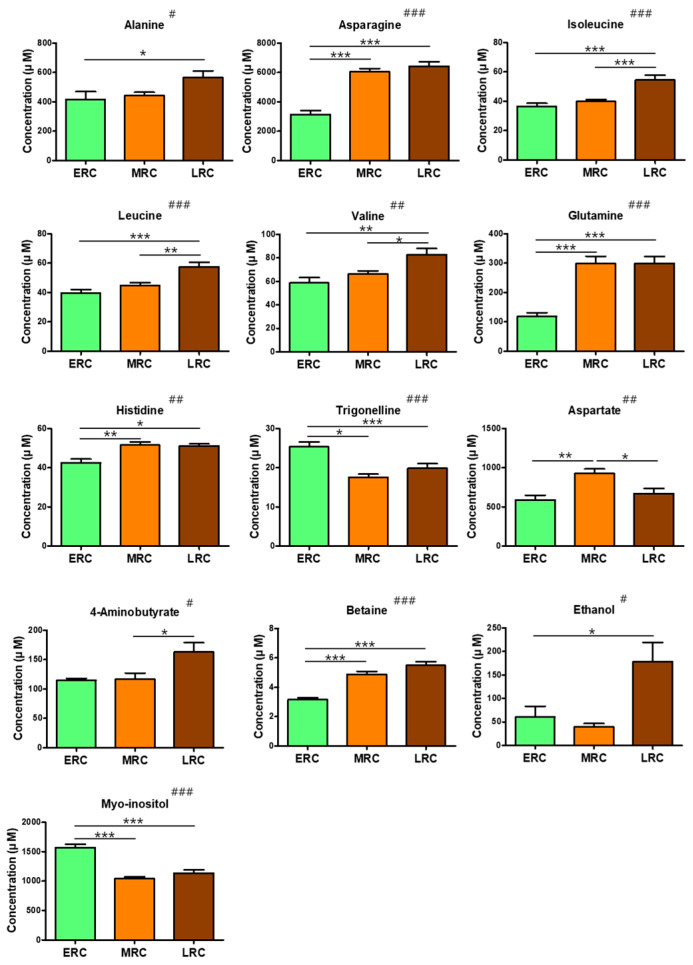
Levels of metabolites in early-ripening cultivars, mid-ripening cultivars and late-ripening cultivars of *C. crenata* chestnut. Kruskal–Wallis tests yielded significant differences determined by Dunn’s multiple comparison post hoc tests using GraphPad Prism 5.0. Significant differences by Kruskal–Wallis tests are represented as # *p* < 0.05, ## *p* < 0.01, and ### *p* < 0.001, and by Dunn’s multiple comparison post hoc tests are represented as * *p* < 0.05, ** *p* < 0.01, and *** *p* < 0.001. Error bars indicate means ± standard deviation. Letters on the horizontal axis represent: ERC, early-ripening cultivar; MRC, mid-ripening cultivar; LRC, late-ripening cultivar.

A Kruskal–Wallis test with Dunn’s multiple comparison post hoc test was used to compare the three groups. Of the 30 metabolites quantified, 13 showed significant differences in levels between the ERC, MRC, and LRC groups (Figure 5). In particular, nine metabolites with significant differences between the three groups had the highest concentrations in the LRC group.

Of the 13 metabolites with significant differences, nine were amino acids, suggesting that the harvest time of *C. crenata* is one of the main factors affecting amino acid levels. A previous study has shown that the levels of free amino acids and sugars in peanuts decline after the early harvest time, plateau during the optimum harvest season, and increase during the late harvest time [41]. The analysis of free amino acids in the stems of *Acanthopanax koreanum* Nakai, harvested in May, July, and September, showed the highest concentrations in those harvested in September [42]. Our results showed that most amino acid levels were high in the LRC group, except those of trigonelline and aspartate.

Betaine, ethanol, GABA, and myo-inositol had significant differences in concentrations between the groups. The level of GABA was significantly higher in the LRC group than in the MRC group, and betaine and ethanol levels were significantly higher in the LRC group than in the ERC group. In contrast, the concentration of myo-inositol was higher in the ERC group than in the LRC group. Myo-inositol oxidizes to d-glucuronic acid and plays a role in the biogenesis of plant cell walls and related structures [43,44]. The isomerization and methylation of myo-inositol forms O-methyl inositol, which participates in stress-related responses and seed product storage [45,46,47,48,49].

## 4. Conclusions

In this study, we determined the metabolite profiles of various cultivars of *C. crenata* to investigate the effects of different origins and harvest times. Metabolite profiling using ^1^H NMR spectroscopy revealed that the levels of primary metabolites in various groups of *C. crenata* were significantly different according to their origin and harvest time. The KN, JN, and JH (origin) groups could be distinguished according to their metabolite profiles, especially JH, which was the most strongly separated group. The significantly different metabolites across the three groups (KN, JN, and JH) included 13 amino acids, two sugars, one organic acid, and three additional compounds. The significantly different metabolites across the three harvest time groups (ERC, MRC, and LRC) included nine amino acids and four other compounds. The concentrations of significantly different metabolites were higher in the LRC group, and myo-inositol was detected at high levels in the ERC group. In particular, significant differences in amino acid concentrations were observed, suggesting that the origin and harvest time of *C. crenata* affect the composition of amino acids. This is the first report to describe different metabolite compositions in *C. crenata* chestnut kernels with different origins and harvest times using ^1^H NMR spectral analysis. Since there have been few metabolomics studies on chestnut kernels before, this study provides information on the metabolites of chestnut kernels. Our results provide information regarding the differences in metabolite profiles associated with various *C. crenata* cultivars and can be used to improve the nutritional or metabolic benefits of *C. crenata* chestnut kernels. These results can help growers and consumers intentionally select chestnut cultivars that satisfy their nutritional and taste needs. This information could also be useful for breeding programs to develop superior *C. crenata* varieties. In addition, although we have only focused on metabolite differences between on cultivars, further study on the genetic and metabolite differences of chestnut cultivars together will provide good information for producing better chestnut cultivars.

## Figures and Tables

**Table 1:** Sample list of *Castanea crenata* chestnut used in this study.

Region	Number of Samples	Cultivar Name	Origin	Crossbreeding	Origin Group	Harvest Time
Chungchengnam-do (latitude 36° N, Longitude 127° E)	5	Okkwang	Korea	*crenata* ^a^	KN	Mid ripening
	5	Jahong	Korea	*crenata* ^a^	KN	Mid ripening
	5	Mipung	Korea	*crenata* ^a^	KN	Late ripening
	5	Juok	Korea	*crenata* hybrid (Gwangjujoyul ^a^ × Okkwang ^a^)	KN	Mid ripening
	3	Pyeonggi	Korea	*crenata* hybrid (Riheiguri × Ginyosi ^b^)	KH	Mid ripening
	4	Daebo	Korea	*crenata* hybrid (Sangmyeon 1 ^a^ × Riheiguri)	KH	Mid ripening
	4	Idea	Korea	*crenata* hybrid (Ibuki × Sandae ^a^)	KH	Late ripening
	5	Otanba	Japan	*crenata* ^b^	JN	Mid ripening
	5	Ginyosi	Japan	*crenata* ^b^	JN	Late ripening
	5	Tanzawa	Japan	*crenata* hybrid (Otomune ^b^ × Taishouwase ^b^)	JN	Early ripening
	5	Tsukuba	Japan	*crenata* hybrid (Ganne ^b^ × Hayadama ^b^)	JN	Mid ripening
	4	Ishizuuchi	Japan	*crenata* hybrid (Ganne ^b^ × Kasaharawase ^b^)	JN	Late ripening
	5	Porotan	Japan	(*crenata* × *bungeana*) × *crenata* hybrid (550-40 × Tanzawa ^b^)	JH	Early ripening
	3	Moriwase	Japan	*crenata* × *bungeana* hybrid (Pyungyangyul × Toyotamawase ^b^)	JH	Early ripening
	4	Riheiguri	Japan	*crenata* × *mollissima* hybrid	JH	Mid ripening
	4	Hyogo57	Japan	*crenata* × *mollissima* hybrid	JH	Mid ripening
	5	Banseki ^c^	Japan	-	-	Late ripening

^a^ The origin of cultivars was Korea only. ^b^ The origin of cultivars was Japan only. ^c^ Since there was no information on crossbreeding, the Banseki cultivar was excluded from the groups classified according to origin.

**Table 2:** Metabolites and ^1^H chemical shifts identified by 800 MHz ^1^H NMR ^a^.

No.	Metabolites	^1^H Chemical Shifts ^b^	Formula
Amino acids			
1	Alanine	1.5 (d), 3.8 (q)	C_3_H_7_NO_2_
2	Arginine	1.7 (m), 1.9 (m), 3.2 (t), 3.8 (t)	C_6_H_14_N_4_O_2_
3	Asparagine	2.8 (q), 2.9 (dd), 3.9 (q), 6.9 (s)	C_4_H_8_N_2_O_3_
4	Aspartate	2.6 (q), 2.8 (dd), 3.8 (dd)	C_4_H_7_NO_4_
5	Glutamate	2.0 (m), 2.1 (m), 2.4 (m), 3.8 (q)	C_5_H_9_NO_4_
6	Glutamine	2.44 (m), 2.12 (m)	C_5_H_10_N_2_O_3_
7	Histidine	3.2 (m), 4.0 (t), 7.1 (s), 7.9 (s)	C_6_H_9_N_3_O_2_
8	Isoleucine	0.9 (t), 1.0 (d), 1.2 (m), 1.5 (m), 2.0 (m), 3.7 (d)	C_6_H_13_NO_2_
9	Leucine	0.9(d), 1.0 (d), 1.7 (m), 1.7 (m), 1.7 (m), 3.7 (q)	C_6_H_13_NO_2_
10	Phenylalanine	3.1 (q), 3.3 (q), 4.0 (q), 7.3 (d), 7.4 (m)	C_9_H_11_NO_2_
11	Threonine	1.3 (d), 3.6 (d), 4.3 (q)	C_4_H_9_NO_3_
12	Trigonelline	8.0 (t), 8.8 (dd), 9.1 (s)	C_7_H_7_NO_2_
13	Tryptophan	7.1 (t), 7.2 (t), 7.3 (s), 7.5 (d), 7.7 (d)	C_11_H_12_N_2_O_2_
14	Tyrosine	3.1 (q), 3.2 (q), 3.9 (q), 6.8 (d), 7.2 (d)	C_9_H_11_NO_3_
15	Valine	1.0 (d), 2.3 (m), 3.6 (d)	C_5_H_11_NO_2_
Organic acids			
16	Citric acid	2.5 (d), 2.7 (d)	C_6_H_8_O_7_;
17	Formic acid	8.4 (s)	CH_2_O_2_
18	Fumaric acid	6.5 (s)	C_4_H_4_O_4_
19	Malic acid	2.4 (q), 2.7 (dd), 4.3 (d)	C_4_H_6_O_5_
20	Malonic acid	3.1 (s)	C_3_H_4_O_4_
21	Succinic acid	2.3 (s)	C_4_H_6_O_4_
Sugars			
22	Fructose	3.5–4.1 (m)	C_6_H_12_O_6_
23	Glucose	3.2 (q), 3.4 (m), 3.5 (q), 3.7 (m), 4.6 (d), 5.2 (d)	C_6_H_12_O_6_
24	Sucrose	3.4 (t), 3.5 (dd), 3.6 (dd), 3.7 (t), 3.8 (m), 4.0 (t), 4.2 (d), 5.4 (d)	C_12_H_22_O_11_
Others			
25	4-Aminobutyrate	1.9 (m), 2.3 (t), 3.0 (t)	C_4_H_9_NO_2_
26	Betaine	3.3 (s), 3.9 (s)	(CH_3_)_3_N^+^ CH_2_COO^−^
27	Choline	3.2 (s), 3.5 (m), 4.0 (m)	C_5_H_14_NO
28	Ethanol	1.18 (t), 3.6 (q)	C_2_H_5_OH
29	Ethanolamine	3.1 (t), 3.8 (t)	C_2_H_7_NO
30	Myo-inositol	3.2 (t), 3.5 (dd), 3.6 (t), 4.0 (t)	C_6_H_12_O_6_

^a^ The chemical shifts were determined at pH 7.0 and expressed as relative values to those of TSP at 0 ppm. ^b^ Letters in parentheses indicate the peak multiplicities: s, singlet; d, doublet; t, triplet; dd, doublet of doublet; tt, triplet of triplet; q, quartet; and m, multiplet.

## Data Availability

The data presented in this study are available within the manuscript and the Supplementary Materials.

## Supplementary Materials

The following supporting information can be downloaded at: https://www.mdpi.com/article/10.3390/foods11091325/s1, Table S1: Metabolite concentrations quantified by ^1^H NMR in *C. crenata* according to geographical origin; Table S2: Metabolite concentrations quantified by ^1^H NMR in *C. crenata* according to harvest time.

## References

[1] Chiocchio I., Prata C., Mandrone M., Ricciardiello F., Marrazzo P., Tomasi P., Angeloni C., Fiorentini D., Malaguti M., Poli F. (2020). Leaves and spiny burs of Castanea Sativa from an experimental chestnut grove: Metabolomic analysis and anti-neuroinflammatory activity. Metabolites.

[2] Tsurunaga Y., Takahashi T. (2021). Evaluation of the Antioxidant Activity, Deodorizing Effect, and Antibacterial Activity of ‘Porotan’Chestnut By-Products and Establishment of a Compound Paper. Foods.

[3] Silva D., Zambon C., Techio V., Pio R. (2020). Floral characterization and pollen germination protocol for Castanea crenata Siebold & Zucc. S. Afr. J. Bot..

[4] Kang M.-J., Shin A.-Y., Shin Y., Lee S.-A., Lee H.-R., Kim T.-D., Choi M., Koo N., Kim Y.-M., Kyeong D. (2019). Identification of transcriptome-wide, nut weight-associated SNPs in Castanea crenata. Sci. Rep..

[5] Korea Statistical Information Service. http://kosis.kr.

[6] Youn U.-Y., Shon M.-S., Kim G.-N., Katagiri R., Harata K., Ishida Y., Lee S.-C. (2016). Antioxidant and anti-adipogenic activities of chestnut (Castanea crenata) byproducts. Food Sci. Biotechnol..

[7] Pereira-Lorenzo S., Ramos-Cabrer A.M., Díaz-Hernández M., Ciordia-Ara M., Ríos-Mesa D. (2006). Chemical composition of chestnut cultivars from Spain. Sci. Hortic..

[8] Tuyen P.T., Xuan T.D., Khang D.T., Ahmad A., Quan N.V., Tu Anh T.T., Anh L.H., Minh T.N. (2017). Phenolic compositions and antioxidant properties in bark, flower, inner skin, kernel and leaf extracts of Castanea crenata Sieb. et Zucc. Antioxidants.

[9] Noh J.-R., Gang G.-T., Kim Y.-H., Yang K.-J., Hwang J.-H., Lee H.-S., Oh W.-K., Song K.-S., Lee C.-H. (2010). Antioxidant effects of the chestnut (Castanea crenata) inner shell extract in t-BHP-treated HepG2 cells, and CCl4-and high-fat diet-treated mice. Food Chem. Toxicol..

[10] Kim M., Lee U., Kim S., Hwang M., Lee M. Comparison of nut characteristics between Korean native chestnut accessions and prevailing cultivars cultivated in Korea. Proceedings of the III International Chestnut Congress 693.

[11] Yamamoto T., Shimada T., Kotobuki K., Morimoto Y., Yoshida M. (1998). Genetic characterization of Asian chestnut varieties assessed by AFLP. Jpn. J. Breed..

[12] Yamamoto T., Tanaka T., Kotobuki K., Matsuta N., Suzuki M., Hayashi T. (2003). Characterization of simple sequence repeats in Japanese chestnut. J. Hortic. Sci. Biotechnol..

[13] Seo D.-J., Chung M.-J., Kim D.-J., You J.-K., Choe M. (2009). Nutritional constituent analysis of Korean chestnuts. J. Korean Soc. Food Sci. Nutr..

[14] Jeong H.-R., Jo Y.-N., Jeong J.-H., Jin D.-E., Song B.-G., Jin Y.-R., Kim M.-J., Lee U., Heo H.-J. (2012). Change in the chemical composition of chestnuts (Castanea crenata) from different periods. Korean J. Food Sci. Technol..

[15] Hall R.D. (2006). Plant metabolomics: From holistic hope, to hype, to hot topic. New Phytol..

[16] Hall R.D. (2007). Food metabolomics: META-PHOR. A new European research initiative. Agro FOOD Industry Hi Tech.

[17] Son H.-S., Hwang G.-S., Kim K.M., Ahn H.-J., Park W.-M., Van Den Berg F., Hong Y.-S., Lee C.-H. (2009). Metabolomic studies on geographical grapes and their wines using 1H NMR analysis coupled with multivariate statistics. J. Agric. Food Chem..

[18] Porzel A., Farag M.A., Mülbradt J., Wessjohann L.A. (2014). Metabolite profiling and fingerprinting of Hypericum species: A comparison of MS and NMR metabolomics. Metabolomics.

[19] Dixon R.A., Gang D.R., Charlton A.J., Fiehn O., Kuiper H.A., Reynolds T.L., Tjeerdema R.S., Jeffery E.H., German J.B., Ridley W.P. (2006). Applications of metabolomics in agriculture. J. Agric. Food Chem..

[20] Calò F., Girelli C.R., Wang S.C., Fanizzi F.P. (2022). Geographical Origin Assessment of Extra Virgin Olive Oil via NMR and MS Combined with Chemometrics as Analytical Approaches. Foods.

[21] Hama J.R., Fitzsimmons-Thoss V. (2022). Determination of Unsaturated Fatty Acids Composition in Walnut (*Juglans regia* L.) Oil Using NMR Spectroscopy. Food Anal. Methods.

[22] Cacciola N.A., Cerrato A., Capriotti A.L., Cavaliere C., D’Apolito M., Montone C.M., Piovesana S., Squillaci G., Peluso G., Laganà A. (2020). Untargeted characterization of chestnut (Castanea sativa Mill.) shell polyphenol extract: A valued bioresource for prostate cancer cell growth inhibition. Molecules.

[23] Cerulli A., Napolitano A., Masullo M., Hošek J., Pizza C., Piacente S. (2020). Chestnut shells (Italian cultivar “Marrone di Roccadaspide” PGI): Antioxidant activity and chemical investigation with in depth LC-HRMS/MSn rationalization of tannins. Food Res. Int..

[24] Jung Y., Lee J., Kim H.K., Moon B.C., Ji Y., Hwang G.-S. (2012). Metabolite profiling of Curcuma species grown in different regions using 1 H NMR spectroscopy and multivariate analysis. Analyst.

[25] Lee M.Y., Moon B.C., Kwon Y.K., Jung Y., Oh T.K., Hwang G.S. (2016). Discrimination of Polygonatum species and identification of novel markers using 1H NMR-and UPLC/Q-TOF MS-based metabolite profiling. J. Sci. Food Agric..

[26] Jung Y., Ha M., Lee J., Ahn Y.G., Kwak J.H., Ryu D.H., Hwang G.-S. (2015). Metabolite profiling of the response of burdock roots to copper stress. J. Agric. Food Chem..

[27] Worley B., Powers R. (2013). Multivariate analysis in metabolomics. Curr. Metab..

[28] Mattila P., Salo-Väänänen P., Könkö K., Aro H., Jalava T. (2002). Basic composition and amino acid contents of mushrooms cultivated in Finland. J. Agric. Food Chem..

[29] Phat C., Moon B., Lee C. (2016). Evaluation of umami taste in mushroom extracts by chemical analysis, sensory evaluation, and an electronic tongue system. Food Chem..

[30] De Vasconcelos M.d.C.B., Nunes F., Viguera C.G., Bennett R.N., Rosa E.A., Ferreira-Cardoso J.V. (2010). Industrial processing effects on chestnut fruits (Castanea sativa Mill.) 3. Minerals, free sugars, carotenoids and antioxidant vitamins. Int. J. Food Sci. Technol..

[31] Fernandes Â., Antonio A.L., Barros L., Barreira J.C., Bento A., Botelho M.L., Ferreira I.C. (2011). Low dose γ-irradiation as a suitable solution for chestnut (Castanea sativa Miller) conservation: Effects on sugars, fatty acids, and tocopherols. J. Agric. Food Chem..

[32] Barreira J., Pereira J.A., Oliveira M., Ferreira I.C. (2010). Sugars profiles of different chestnut (Castanea sativa Mill.) and almond (Prunus dulcis) cultivars by HPLC-RI. Plant Foods Hum. Nutr..

[33] Suárez M.H., Galdón B.R., Mesa D.R., Romero C.D., Rodríguez E.R. (2012). Sugars, organic acids and total phenols in varieties of chestnut fruits from Tenerife (Spain). Food Nutr. Sci..

[34] Ribeiro B., Rangel J., Valentão P.c., Andrade P.B., Pereira J.A., Bölke H., Seabra R.M. (2007). Organic acids in two Portuguese chestnut (Castanea sativa Miller) varieties. Food Chem..

[35] Servillo L., Giovane A., Casale R., Balestrieri M.L., Cautela D., Paolucci M., Siano F., Volpe M.G., Castaldo D. (2016). Betaines and related ammonium compounds in chestnut (Castanea sativa Mill.). Food Chem..

[36] Du C., Chen W., Wu Y., Wang G., Zhao J., Sun J., Ji J., Yan D., Jiang Z., Shi S. (2020). Effects of GABA and vigabatrin on the germination of Chinese chestnut recalcitrant seeds and its implications for seed dormancy and storage. Plants.

[37] Bouche N., Fromm H. (2004). GABA in plants: Just a metabolite?. Trends Plant Sci..

[38] Kinnersley A.M., Turano F.J. (2000). Gamma aminobutyric acid (GABA) and plant responses to stress. Crit. Rev. Plant Sci..

[39] Bouché N., Lacombe B.t., Fromm H. (2003). GABA signaling: A conserved and ubiquitous mechanism. Trends Cell Biol..

[40] Ramesh S.A., Tyerman S.D., Gilliham M., Xu B. (2017). γ-Aminobutyric acid (GABA) signalling in plants. Cell. Mol. Life Sci..

[41] Oupadissakoon C., Young C.T., Giesbrecht F.G., Perry A. (1980). Effect of location and time of harvest on free amino acid and free sugar contents of Florigiant peanuts. Peanut Sci..

[42] Jwa C.-S., Yang Y.-T., Koh J.-S. (2000). Changes in free sugars, organic acids, free amino acids and minerals by harvest time and parts of Acanthopanax koreanum. Appl. Biol. Chem..

[43] Loewus F.A., Loewus M.W. (1983). Myo-inositol: Its biosynthesis and metabolism. Annu. Rev. Plant Physiol..

[44] Loewus F. (2012). Biogenesis of Plant Cell Wall Polysaccharides.

[45] Horbowicz M., Obendorf R.L. (1994). Seed desiccation tolerance and storability: Dependence on flatulence-producing oligosaccharides and cyclitols—Review and survey. Seed Sci. Res..

[46] Bohnert H.J., Jensen R.G. (1996). Strategies for engineering water-stress tolerance in plants. Trends Biotechnol..

[47] Obendorf R.L. (1997). Oligosaccharides and galactosyl cyclitols in seed desiccation tolerance. Seed Sci. Res..

[48] Peterbauer T., Puschenreiter M., Richter A. (1998). Metabolism of galactosylononitol in seeds of Vigna umbellata. Plant Cell Physiol..

[49] Peterbauer T., Richter A. (1998). Galactosylononitol and stachyose synthesis in seeds of adzuki bean: Purification and characterization of stachyose synthase. Plant Physiol..

