# Supplementary material for: Metabolite Profiling of Chestnut (Castanea crenata) According to Origin and Harvest Time Using 1H NMR Spectroscopy
Source: Foods. 2022 May 2;11(9):1325. doi: 10.3390/foods11091325 (PMC9099845; doi:10.3390/foods11091325)
Supplement: Supplementary file 1 [file foods-11-01325-s001.zip › foods-1691111-supplementary.pdf]

Table S1. Metabolite concentrations quantified by ^{1}H NMR in *C. crenata* according to geographical origin.

No.	Metabolites	Concentrations (μM)			
		KN	KH	JN	JH
		Amino acids			
1	Alanine	400.95 ± 150.71	495.90 ± 153.04	515.16 ± 221.64	488.23 ± 164.46
2	Arginine ###	423.40 ± 231.34	798.21 ± 221.63	745.54 ± 212.76	482.15 ± 182.56
3	Asparagine ##	6090.51 ± 1533.17	5860.81 ± 1273.54	6148.09 ± 1964.97	4200.75 ± 1597.84
4	Aspartate ###	1046.96 ± 279.42	851.21 ± 204.73	598.27 ± 241.20	691.12 ± 465.05
5	Glutamate	1854.95 ± 485.77	1823.17 ± 256.60	2058.50 ± 246.94	1635.99 ± 612.96
6	Glutamine ###	323.08 ± 128.71	444.95 ± 104.39	229.04 ± 144.90	149.95 ± 45.08
7	Histidine ###	52.48 ± 6.66	54.44 ± 6.39	53.68 ± 7.73	38.01 ± 6.13
8	Isoleucine ###	45.33 ± 9.29	41.75 ± 8.28	44.70 ± 6.34	30.71 ± 3.78
9	Leucine ###	53.55 ± 9.87	46.94 ± 14.86	48.40 ± 8.58	31.76 ± 5.80
10	Phenylalanine #	25.65 ± 7.81	22.80 ± 8.34	32.44 ± 12.80	20.38 ± 10.31
11	Threonine ###	192.46 ± 28.42	353.03 ± 228.98	269.90 ± 169.13	143.26 ± 24.53
12	Trigonelline ###	16.70 ± 3.67	16.95 ± 5.36	19.35 ± 6.94	23.74 ± 3.48
13	Tryptophan ###	18.76 ± 2.40	18.81 ± 5.01	19.17 ± 3.82	9.97 ± 3.98
14	Tyrosine #	26.81 ± 6.26	23.83 ± 6.86	27.49 ± 5.27	21.29 ± 7.67
15	Valine ###	69.49 ± 20.05	67.65 ± 13.19	75.11 ± 12.13	49.92 ± 7.18
		Organic acids			
16	Citric acid ###	555.38 ± 159.67	419.14 ± 82.29	487.53 ± 110.77	383.65 ± 223.16
17	Formic acid	2.13 ± 1.23	2.09 ± 0.61	2.21 ± 1.23	2.03 ± 1.06
18	Fumaric acid	11.77 ± 3.37	13.39 ± 2.54	12.55 ± 2.69	14.41 ± 4.91
19	Malic acid	2450.04 ± 629.01	1913.03 ± 374.65	2265.86 ± 477.33	2304.86 ± 359.54
20	Malonic acid	7.43 ± 4.42	11.88 ± 3.68	7.96 ± 3.23	7.73 ± 3.78
21	Succinic acid	3.05 ± 2.58	2.29 ± 1.16	3.84 ± 2.18	5.41 ± 6.17
		Sugars			
22	Fructose #	2481.09 ± 743.13	1904.87 ± 351.77	1910.05 ± 553.90	2301.63 ± 767.34
23	Glucose #	1435.22 ± 470.19	1123.82 ± 315.43	1090.22 ± 481.01	1424.48 ± 567.18
24	Sucrose	38,942.50 ± 3626.07	35,617.87 ± 2012.83	42,280.26 ± 5730.92	40,083.06 ± 5472.51
		Others			
25	4-Aminobutyrate ##	104.48 ± 80.77	107.25 ± 30.32	142.30 ± 71.67	148.39 ± 34.62
26	Betaine ###	4.84 ± 1.09	5.05 ± 0.87	5.20 ± 1.63	3.42 ± 0.61
27	Choline ##	193.11 ± 23.99	206.65 ± 32.93	204.38 ± 25.42	174.05 ± 25.45
28	Ethanol	48.70 ± 59.93	20.49 ± 7.97	104.95 ± 137.68	63.08 ± 72.94
29	Ethanolamine	75.12 ± 14.15	79.24 ± 7.19	80.02 ± 8.62	76.23 ± 10.84
30	Myo-inositol	1122.50 ± 138.57	804.03 ± 98.90	1193.99 ± 295.09	1332.46 ± 300.84

The results are represented as mean ± standard deviations. Significant differences by Kruskal–Wallis tests are represented as # $p < 0.05$, ## $p < 0.01$, and ### $p < 0.001$, among KN, JN, and JH.

Table S2. Metabolite concentrations quantified by ^{1}H NMR in *C. crenata* according to harvest time.

No.	Metabolites	Concentrations (μM)		
		**Early-Ripening**	**Mid-Ripening**	**Late-Ripening**
		Amino acids		
1	Alanine #	416.73 ± 192.69	443.77 ± 137.69	566.83 ± 211.61
2	Arginine	473.13 ± 127.39	602.33 ± 268.38	601.40 ± 321.80
3	Asparagine ###	3142.49 ± 965.63	6047.87 ± 1398.61	6434.87 ± 1426.86
4	Aspartate ##	587.65 ± 220.06	927.44 ± 356.20	672.12 ± 304.87
5	Glutamate	1868.03 ± 423.65	1750.57 ± 460.85	1984.87 ± 403.17
6	Glutamine ###	118.94 ± 44.82	299.11 ± 156.49	299.38 ± 113.53
7	Histidine ##	42.50 ± 7.00	51.60 ± 10.40	51.09 ± 5.15
8	Isoleucine ###	36.58 ± 7.52	39.82 ± 8.82	54.53 ± 15.77
9	Leucine ###	39.70 ± 8.57	44.71 ± 13.36	57.65 ± 14.12
10	Phenylalanine	34.78 ± 14.51	23.94 ± 9.91	27.73 ± 9.57
11	Threonine	170.50 ± 31.62	228.00 ± 143.71	269.38 ± 174.03
12	Trigonelline ###	25.37 ± 4.52	17.53 ± 5.20	19.86 ± 5.64
13	Tryptophan	15.41 ± 5.90	16.65 ± 5.63	19.58 ± 3.81
14	Tyrosine	28.05 ± 4.12	24.37 ± 7.55	28.41 ± 8.46
15	Valine ##	58.69 ± 16.72	66.39 ± 16.74	82.79 ± 25.01
		Organic acids		
16	Citric acid	389.57 ± 142.48	483.10 ± 167.23	511.63 ± 140.80
17	Formic acid	2.28 ± 0.83	2.05 ± 1.27	2.03 ± 1.16
18	Fumaric acid	14.62 ± 5.18	12.51 ± 3.26	13.74 ± 3.80
19	Malic acid	2222.93 ± 319.07	2232.46 ± 495.65	2749.96 ± 915.31
20	Malonic acid	8.31 ± 4.10	8.36 ± 3.93	7.83 ± 4.02
21	Succinic acid	4.56 ± 2.32	3.73 ± 4.48	3.25 ± 1.29
		Sugars		
22	Fructose	2301.48 ± 545.47	2099.97 ± 740.05	2292.27 ± 669.02
23	Glucose	1289.63 ± 361.19	1293.79 ± 574.95	1261.77 ± 415.81
24	Sucrose	40,047.40 ± 6005.85	40,027.84 ± 5056.73	40,597.93 ± 5344.57
		Others		
25	4-Aminobutyrate #	114.85 ± 12.55	117.04 ± 64.57	163.31 ± 74.49
26	Betaine ###	3.15 ± 0.46	4.88 ± 1.26	5.49 ± 1.20
27	Choline	181.60 ± 21.92	202.16 ± 31.89	196.92 ± 28.05
28	Ethanol #	60.49 ± 82.64	39.80 ± 44.27	178.55 ± 194.64
29	Ethanolamine	74.75 ± 9.78	79.16 ± 12.04	76.70 ± 7.46
30	Myo-inositol ###	1571.94 ± 199.47	1042.87 ± 185.20	1137.87 ± 263.95

The results are represented as mean ± standard deviations. Significant differences by Kruskal–Wallis tests are represented as # $p < 0.05$, ## $p < 0.01$, and ### $p < 0.001$, among early-ripening, mid-ripening, and late-ripening varieties.
